# Perceptual difference of smile aesthetics between 2-dimensional photographs and 3-dimensional dentofacial images: a cross-sectional study

**DOI:** 10.1186/s12903-023-02798-2

**Published:** 2023-02-16

**Authors:** Zhuoxing Xiao, Gui Chen, Yijiao Zhao, Yong Wang, Yan Gu

**Affiliations:** 1 National Center for Stomatology & National Clinical Research Center for Oral Disease & National Engineering Research Center of Oral Biomaterials and Digital Medical Devices & Beijing Key Laboratory of Digital Stomatology, Key Laboratory of Digital Stomatology & Research Center of Engineering and Technology for Computerized Dentistry Ministry of Health & NMPA Key Laboratory for Dental Materials, Department of Orthodontics, Peking University School and Hospital of Stomatology, No. 22 Zhongguancun South Avenue, Haidian District, Beijing, 100081 People’s Republic of China; 2 National Center for Stomatology & National Clinical Research Center for Oral Disease % National Engineering Research Center of Oral Biomaterials and Digital Medical Devices & Beijing Key Laboratory of Digital Stomatology, Key Laboratory of Digital Stomatology & Research Center of Engineering and Technology for Computerized Dentistry Ministry of Health & NMPA Key Laboratory for Dental Materials, Center of Digital Dentistry, Peking University School and Hospital of Stomatology, No. 22 Zhongguancun South Avenue, Haidian District, Beijing, 100081 People’s Republic of China

**Keywords:** 2D photograph, 3D dentofacial image, Smile, Aesthetics

## Abstract

**Background:**

The aim of this study was to compare the perceptual difference of smile aesthetics between 2D photographs and 3D dentofacial images as perceived by orthodontists and graduate students.

**Methods:**

Forty-eight subjects finished orthodontic treatment were recruited with 2D photographs of frontal, oblique and lateral views as well as 3D dentofacial images. Twelve senior orthodontists and 13 postgraduate students were asked to rate the 2D and 3D smile simulations based on visual analog scale (VAS) and to vote for smile features that affect the attractiveness of smile. At the end, they completed a questionnaire about their views on different smile simulations. Wilcoxon signed-rank, Bland–Altman analysis, and multiple linear regression were used to compare the ratings and votes of smile perception between raters and between records.

**Results:**

Orthodontists and postgraduate students rated smile consistently with 2D photographs, while orthodontists tended to give a higher rate for unattractive smiles and a lower rate for attractive smiles with 3D dentofacial images. The 3D dentofacial images were rated significantly lower than 2D photographs and the voting of most of the smile features showed significant negative main effect on VAS scores, while the effect of demographic characteristics of raters, voting on visible width of upper dentition and buccal corridor was not significant. In addition, a significant negative main effect of commissure and facial profile was found on the rating discrepancy between 2D and 3D images.

**Conclusions:**

Senior orthodontists tend to perceived 3D images more conservatively in smile evaluation. 3D dentofacial images were rated lower than 2D photographs and most of the smile features affect the aesthetic perception of smile. The perceptual difference of commissure and facial profile contributed to the lower ratings in 3D dentofacial images.

**Supplementary Information:**

The online version contains supplementary material available at 10.1186/s12903-023-02798-2.

## Background

Enhancement of facial and dental beauty is an elemental goal of prosthodontic and orthodontic treatment [1, 2]. Orthodontic treatment is required prior to certain restorative treatment to optimize the aesthetic and functional goals. Patients can benefit from the expertise of different disciplines for the interdisciplinary collaboration of compiling a complete problem list and developing a treatment plan agreed upon [3, 4]. Therefore, it is essential to understand the rules of smile perception between and among disciplines and patients to reduce cognitive deviation of the aesthetic treatment goals.

Smile is the most influential part of facial aesthetics compared to the eyes, chin and nose [5]. The perception of smile is a complex phenomenon that affected by biologic, psychologic and social factors [6]. Previous studies have shown that the perception of smile aesthetics is affected by many factors such as gender, age, occupation and education level [7–10]. Sarver et al.[11, 12] suggested that the records of smiles should include frontal, lateral and oblique views. Three-quarter view of face and smiles are of great concern in the evaluation of facial aesthetics [13]. In addition, the different viewing angles affect the aesthetic perception and the measurements of smiles [14, 15]. Therefore, 3-dimensional digital technology has been widely used in the evaluation of facial aesthetics [16–18]. However, the 3D lip-tooth relationships have rarely been studied due to the imaging natures of 3D facial scans that the shape of teeth cannot be well restored for the reflective surfaces [19, 20].

The integration of digital technologies of intraoral scanners and computer aided design software programs produce virtual diagnostic waxing for restorative treatment planning [21–23]. 3D dentofacial image, integrated with 3D facial image and digital waxing, is an effective diagnostic record of patient [22–24]. A preliminary study showed that the 3D dentofacial image is accurate in simulation of the 3D lip-tooth relationship [25]. As a result, the 3-dimensional shape of the smile can be simulated vividly with more details for smile evaluation.

Several studies have compared the perceptual differences in smile evaluation between 2 and 3D simulations. They found that the dimensions of records can influence the perception of smile aesthetics [26–28]. However, it remains unclear how do smile features implement their combined effect on the perceptual difference between traditional 2D photographs and 3D dentofacial images in smile evaluation.

The aim of this study was to compare the perceptual difference of smile aesthetics between 2D photographs and 3D dentofacial images as perceived by orthodontists and graduate students, and to examine factors that can affect smile perceptions. The null hypothesis was that the aesthetic perception of smile is not affected by the dimensional type of images, the occupational type of evaluators, or the perception of a single smile feature.

## Methods

The research protocol was approved by the biomedical ethics committee of human participants of Peking University School and Hospital of Stomatology (PKUSSIRB-201839148). Twenty-nine females (mean age: 20.3 ± 5.0 years) and 19 males (mean age: 21.3 ± 5.0 years) were enrolled in this study. Inclusion criteria: (1) completed orthodontic treatment from November 2018 to September 2019; (2) all teeth were aligned and anterior teeth of upper and lower dentition were well preserved. Exclusion criteria: (1) developmental or traumatic facial malformation or disorder of facial nerve; (2) history of maxillofacial or aesthetic surgery; (3) missing teeth (except for wisdom teeth or premolars extracted for orthodontic treatment); (4) malformations of teeth, enamel defects, caries, or restorations; and (5) progressive periodontal disease. All patients agreed to participate in the study and signed an informed consent form.

**Fig. 1:**
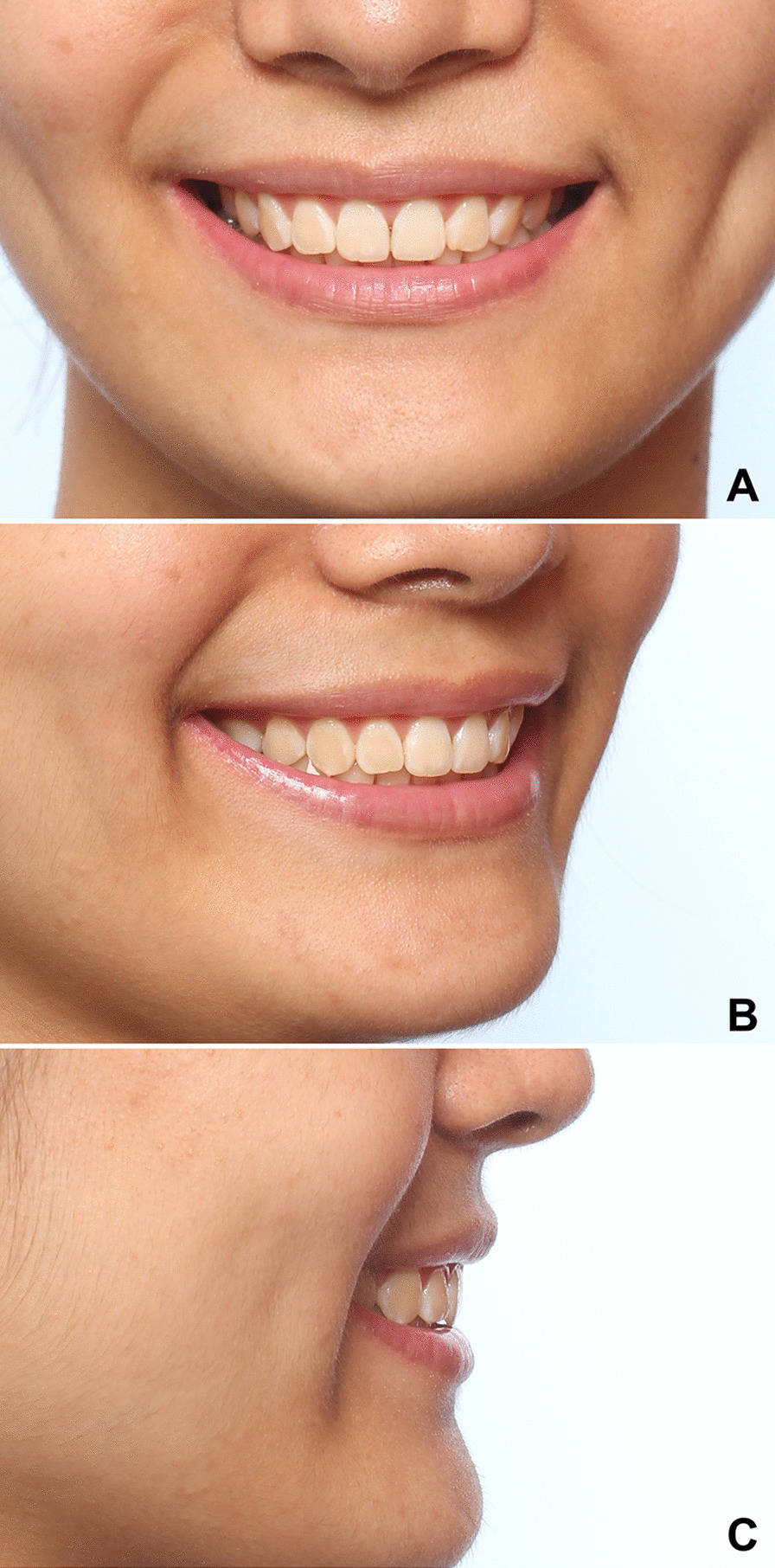
2D photograph of frontal, oblique, and lateral views. **A** Frontal view; **B** oblique view; **C** lateral view

Subjects were adjusted in the natural head position by looking at their eyes in a mirror. They were guided by a verbal directive in order to show a big smile. They were asked to give a smile as big as they can while saying “7” or “cheese”. A Canon EOS 60D camera (Canon, Tokyo, Japan) with a 60-mm F/2.8 macro lens (Canon) was used to record the frontal, oblique and lateral views of smiles (Fig. 1). Subjects were asked to sit at the distance of 1.5 m from the camera. The height of the camera is adjusted to mouth level for each subject. 3D smile images were captured with the 3D optical FaceSCAN3D system (3D-shape, Erlangen, Germany). A total of two 3D facial images were taken, one of which was recorded under the same conditions as the 2D smile image, and the other was taken with the buccal surface of the upper dentition exposed by using a cheek retractor. All the 2D photographs and 3D facial images of smile were taken by the same operator. Each group of images were taken consecutively for 5 times, and the 2D and 3D images with the broadest smile and the highest consistency among images were selected for subsequent evaluation. The 3D digital dentition models were obtained with a 3D scanner (R900; 3Shape, Copenhagen, Denmark).

The integration of 3D dentofacial smile images takes 2 steps with the regional registration function in the Geomagic Studio software (ver. 2014; Geomagic International, NC, USA). Firstly, the digital upper and lower dentitions were superimposed on the 3D facial image with cheek retractor based on the labial surface of the upper dentition from canine to canine. Second, the 3D smile image was superimposed on the 3D facial image with cheek retractor based on the stable area of the forehead and the root of the nose. After the removal of the 3D facial image with cheek retractor, the 3D dentofacial image in the smiling state was reconstructed for further evaluation. The procedure of the integration method of the 3D dental smile image can be seen in detail in the Additional file 1. The accuracy of the integration method in the anterior dental region is within 0.5 mm on all sides of three dimensions [24].

**Fig. 2:**
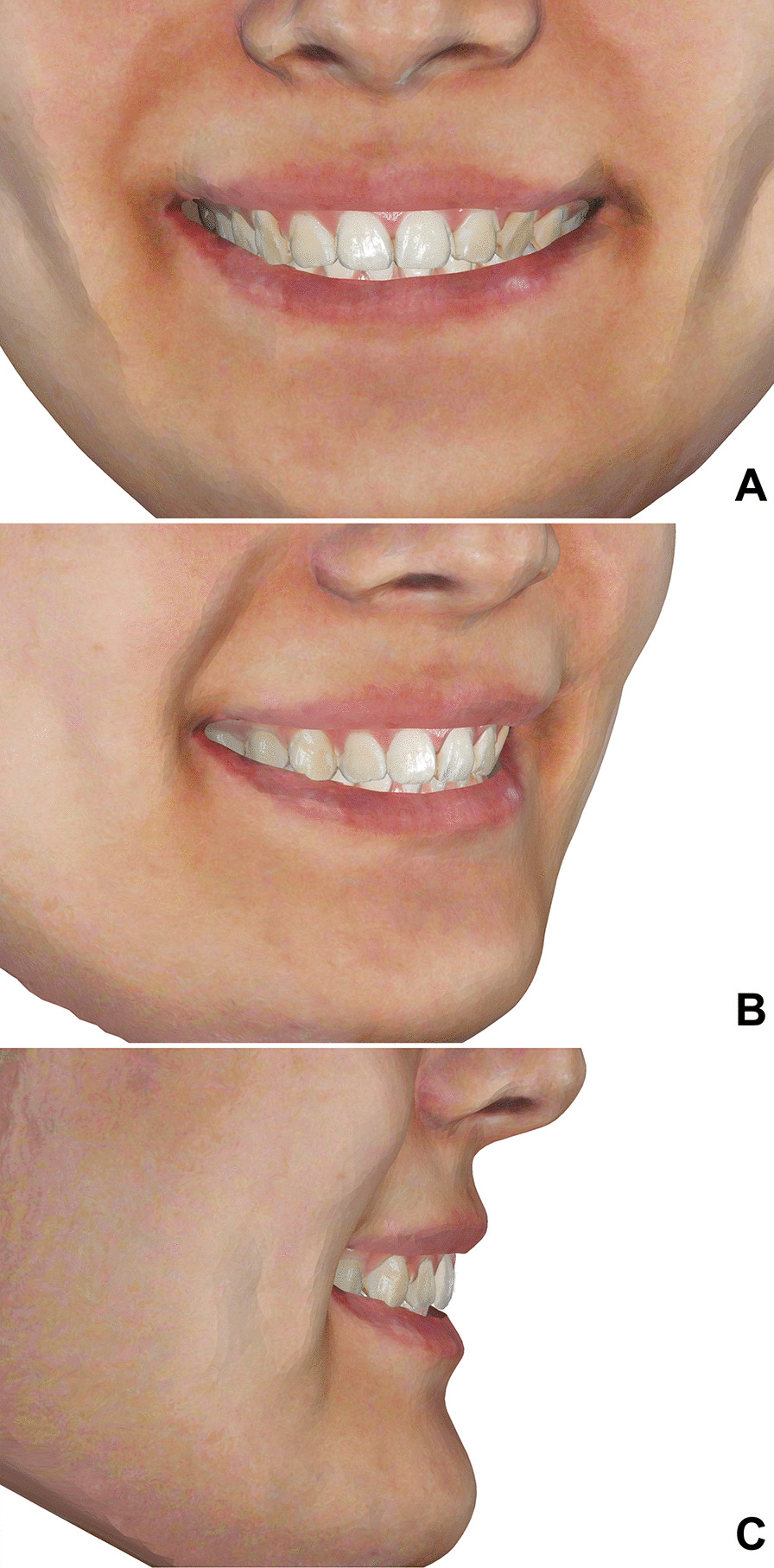
3D dentofacial smile image. **A** Frontal view; **B** oblique view; **C** lateral view

To reduce the interference of other factors on the aesthetic evaluation, all images were unified with the image editing software Photoshop (Version CC; Adobe, San Jose, CA, USA). Spots, scars, and other skin features were removed, and all the 2D and 3D smile images were standardized with the range from the tip of the nose to the chin (Fig. 1, 2). Intraoral photos of frontal and lateral occlusion were collected for each patient. After the adjustment for brightness in Photoshop, the intraoral photos were pasted onto the digital dental model using a point-to-point mapping method in the Geomagic software to simulate a more realistic color of teeth and gum.

Twelve orthodontists (3 males and 9 females) and 13 postgraduate students (3 males and 10 females) were recruited as raters for smile evaluation. The orthodontist group (mean age: 40.0 ± 5.3 years) was defined as full-time faculty members with more than 10 years of clinical experience of Peking University School and Hospital of Stomatology. The postgraduate student group (mean age: 28.2 ± 0.7 years) have an average of 3 years clinical experience in orthodontics department of the same college. Each image was evaluated with two questions. Question 1: “Please evaluate the overall attractiveness of the smile with the VAS.” The VAS from left to right (0–100) represents the least attractive to the most attractive smile. Question 2 is a multiple choices question: "Please choose 1–3 features that have the most negative impact on smile attractiveness." Items of smile features and their definitions are shown in Table 1.

**Table 1:** Smile features for the evaluation of smile aesthetics

Smile features	Definition
Smile pattern	Integral shape of the upper and lower lips when smiling
Commissure	Symmetry of the corner of the mouth when smiling
Dentition	Shape and contour of teeth and gingiva of upper and lower dentition
Visible width of upper dentition	Exposure of the upper dentition in transversal dimension
Buccal corridor	Space between the distal aspect of the canine and the respective commissure
Midline	Deviation of the midline between the upper and lower dentition and lip
Gingival exposure	Vertical gingiva display of the upper dentition
Maxillary incisor exposure	Vertical display of the upper incisors
Mandibular incisor exposure	Vertical display of the lower incisors
Smile arc	Coordination of the curvature of upper incisal edge to the superior border of lower lip
Facial profile	Coordination of the tip of the nose, lip and chin in lateral view
Inclination of incisors	Labial inclination of upper and lower incisors

2D photographs and 3D dentofacial images were displayed on a 39.6-cm screen (resolution 1920 × 1080) of a laptop computer (Legion Y7000 Laptop; Lenovo). Two-dimensional images of smiles were evaluated first. Forty-eight 2D photographs were displayed in form of slides in a random order. Twenty seconds were set for the evaluation process of each image. The female samples were evaluated first, followed by the male samples. After 3 weeks, the corresponding forty-eight 3D smile images were evaluated in the same procedure as the 2D photographs. In order to standardize the evaluation process and ensure that every aspect of the 3D smile image can be observed evenly, the 3D dentofacial images were displayed in the form of video [28]. Each 3D image was converted into video format in which the images will rotate about 180° around the Y axis. The rotation was repeated 3 times within 20 s for smile assessment which is showed in more detail with an additional movie file (see Additional file 2). At the end, evaluators needed to complete a questionnaire about their views on the 3D dentofacial image of smile. Two weeks later, 10 smile images of each type of record were selected randomly for repeated measurement.

### Statistical analyses

The intraclass correlation coefficient (ICC) was used to analyze the retest consistency of VAS scores for smile aesthetics [29]. Bland–Altman analysis was used to determine the agreement of VAS scores between orthodontists and postgraduate students. Wilcoxon signed-rank test were used to compare the ratings and votes between 2D photographs and 3D dentofacial images. To further investigate the combined effect of image type, population group and smile features on the VAS scores of smile aesthetics, a multiple linear regression was conducted with image type, population group and image × population interaction, as well as the number of votes for each smile feature as dependent variables. In addition, to determine the related factors of the rating differences between 2 and 3D images, a multiple linear regression on the rating differences (2D vs. 3D) was established with population group and the voting differences of each smile feature as dependent variables. SPSS software (version 21.0; IBM, Armonk, NY) was used for the statistical analyses. The significance level (α) was set at 0.05 and the Benjamini–Hochberg false discovery rate (FDR) was used for the adjustment of *p* values.

G*power version 3.1.9.2 software was used to determine the sample size by a power analysis. We selected from a t-tests family with Wilcoxon signed-rank test (matched pairs) for comparison of VAS scores and votes between 2D photographs and 3D dentofacial images. Under the condition that the effect size was 0.5 and the two-tail significance level (α) was 0.05, the final sample size of 48 subjects was enough to provide a power above 0.9.

## Results

The ICCs (two-way random, single measures) of the retest consistency of 2D photographs and 3D dentofacial images were 0.862 (0.827–0.891) and 0.632 (0.576–0.682), indicating a good to excellent reproducibility of the VAS scores when assessing smile aesthetics.

**Fig. 3:**
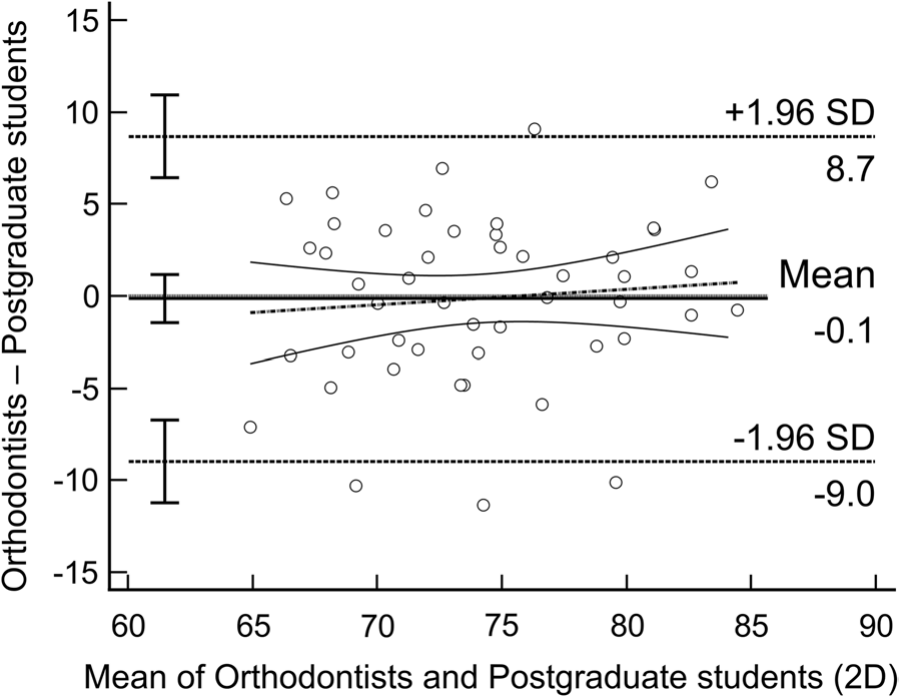
Bland–Altman plots of VAS scores between orthodontists and postgraduate students in 2D photographs

**Fig. 4:**
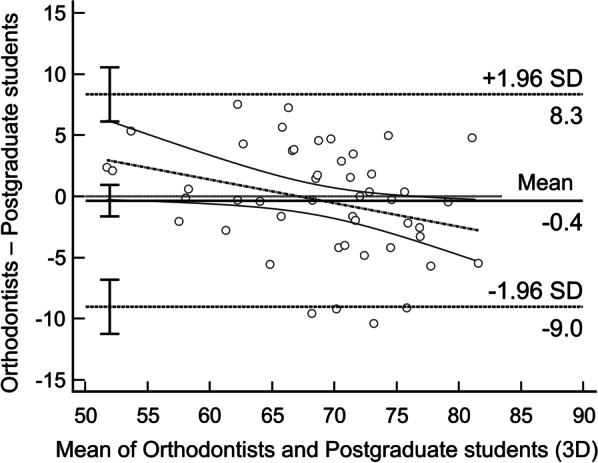
Bland–Altman plots of VAS scores between orthodontists and postgraduate students in 3D dentofacial images

Bland–Altman analysis (Fig. 3, 4) compares the VAS scores across population groups. For both 2D and 3D images, 4/48 (8.3%) points were outside the limits of agreement (LoAs), and 0–1/48 (0–2.1%) points were outside the 95% CI of LoAs when the sampling error was considered. The 95% CI of LoAs were within the 20% clinically acceptable arbitrary limit. In addition, orthodontists and postgraduate students rated smiles consistently across different levels of aesthetics when using 2D photographs. However, orthodontists tended to give a higher rate to the unattractive smiles while they tended to give a lower to the attractive smiles with 3D dentofacial images, revealing that orthodontists perceived the 3D images more conservatively compared with postgraduate students.

**Fig. 5:**
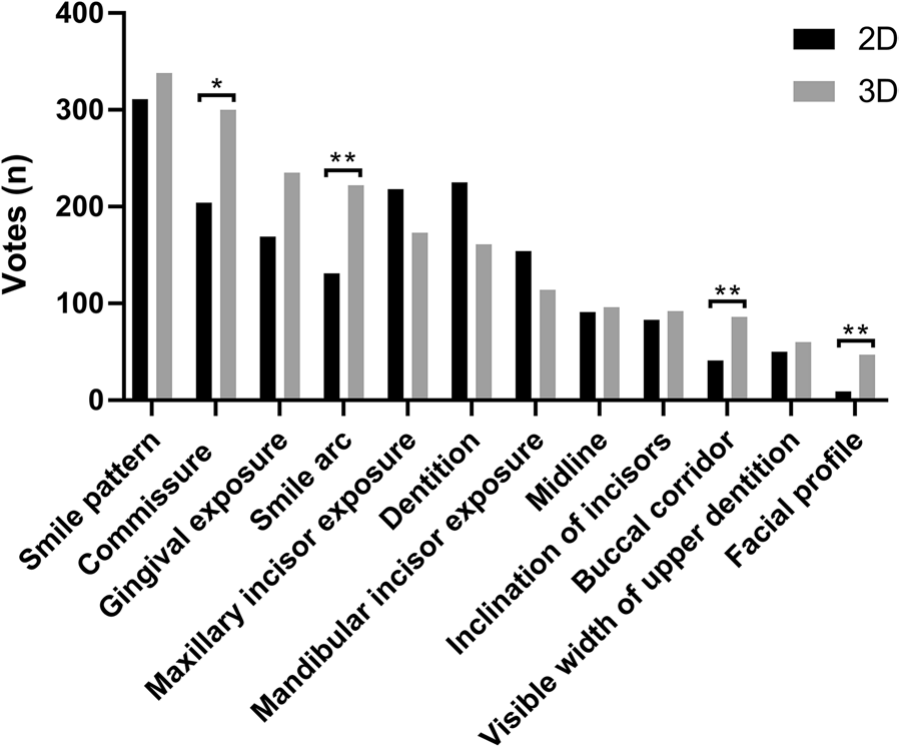
Comparison of votes for smile features between 2D photographs and 3D dentofacial images. (*p < 0.05, **p < 0.001)

For both orthodontists and postgraduate students, 3D dentofacial images were rated lower than 2D photographs, with 95%CI median difference of VAS scores from − 7.190 to − 2.770 (Wilcoxon signed-rank test, *p* < 0.001). In addition, Fig. 5 shows the voting differences of each smile feature between 2D photographs and 3D dentofacial images. 3D images received more votes on facial profile, buccal corridor, smile arc and the symmetry of commissure for affecting smile aesthetics (Wilcoxon signed-rank test, *p* < 0.05).

The image type, population group and their interaction, as well as the votes of each smile features were included as independent variables in the multivariate linear regression of VAS scores (adjusted R square = 0.557). As shown in Table 2, the image type had a significant main effect on the overall VAS scores of smiles, with the scores of 3D images were lower than 2D photographs. No significant main effect was found for population and image × population. In addition, most of the smile features had a significant negative main effect on the overall aesthetic scores of smiles, except for the visible width of upper dentition and the buccal corridor. According to the standardized regression coefficient values, the gingival exposure, upper tooth exposure height, profile and smile pattern were the most influential factors for smile aesthetics.

**Table 2:** Coefficients for multiple linear regression of VAS scores on image type, population group, image × population interaction, and the votes of smile features

Variables	Unstandardized coefficients	Std error	Standardized coefficients	*p*	FDR correction
Gingival exposure	− 11.753	1.461	− 0.430	1.24E−13	1.87E−12**
Maxillary incisor exposure	− 12.903	1.915	− 0.388	2.19E−10	1.64E−09**
Facial profile	− 43.948	6.971	− 0.355	2.26E−09	1.13E−08**
Smile pattern	− 11.422	1.919	− 0.313	1.40E−08	5.24E−08**
Dentition	− 11.940	2.309	− 0.293	6.28E−07	1.89E−06**
Commissure	− 7.501	1.726	− 0.247	2.35E−05	5.87E−05**
Midline	− 10.961	3.064	− 0.180	4.48E−04	9.59E−04**
Mandibular incisor exposure	− 6.671	1.977	− 0.177	9.12E−04	.002*
Smile arc	− 7.402	2.651	− 0.148	.006	.010*
Inclination of incisors	− 8.716	3.189	− 0.151	.007	.010*
Image type	− 2.463	1.074	− 0.176	.023	.031*
Visible width of upper dentition	− 9.263	5.908	− 0.085	.119	.148
population	1.582	1.036	0.113	.128	.148
Buccal corridor	− 4.401	4.685	− 0.049	.349	.374
Image × population	− 0.454	1.414	− 0.028	.748	.748

*p < 0.05, **p < 0.001

A multiple linear regression model of VAS discrepancy between records (Table 3) was established with the population group and voting differences of smile features as independent variables (adjusted R-square = 0.297), which revealed a significant negative main effect of the voting difference of commissure and facial profile. Considering that the commissure and facial profile received more votes in 3D images for affecting smile aesthetics, the lower ratings of 3D images could be explained by more diagnostic information to the commissure and facial profile.

**Table 3:** Coefficients of the multiple linear regression for the differences of VAS scores (2D versus 3D) on population group and voting differences on smile features

Variables	Unstandardized coefficients	Std error	*p*	FDR correction
Commissure	− 8.660	2.771	.002	.018*
Facial profile	− 30.043	9.726	.003	.018*
Gingival exposure	− 7.404	3.075	.018	.065
Dentition	− 9.051	3.832	.021	.065
Visible width of upper Dentition	− 19.537	8.771	.029	.065
Maxillary incisor exposure	− 10.600	4.796	.030	.065
Midline	− 8.637	4.422	.054	.101
Smile pattern	− 5.876	3.285	.077	.126
Smile arc	− 5.564	3.842	.151	.219
Inclination of incisors	− 6.443	6.895	.353	.459
Mandibular incisor exposure	2.490	3.647	.497	.587
Population	0.847	1.483	.569	.617
Buccal corridor	− 3.743	7.521	.620	.620

*p < 0.05

The reviews from raters on traditional 2D photographs and 3D dentofacial images after the evaluation process are showed in Table 4. Fifty percent of the orthodontists and 92% of the postgraduate students believed 3D images can provide more diagnostic information of smile compared with 2D photographs. About 33% orthodontists considered 3D images to be more difficult to master, while only 8% postgraduate students hold the same view. Meanwhile, 42% orthodontists and 62% postgraduate students were more confident with 3D records in smile evaluation. Overall, 50% orthodontists and 69% postgraduate students believed that it is worthy to use 3D images, and none of them felt that 3D images were unnecessary in smile evaluation.

**Table 4:** Qualitative assessment of 2D photographs and 3D dentofacial images

	Orthodontists (%)			Postgraduate students (%)		
	Yes	Indifferent	No	Yes	Indifferent	No
Do you think 3D dentofacial images of smile can provide more diagnostic information?	50	42	8	92	8	0
Do you think 3D dentofacial images of smile are easier to use than 2D photograph?	17	50	33	23	69	8
Are you more confident with 3D records in the evaluation of smile aesthetics?	42	50	8	62	31	8
Do you think it is worthy to use 3D images for the evaluation of smile aesthetics?	50	50	0	69	31	0

Values are present as the percentage of raters agreed with the opinions

## Discussion

The alignment of teeth and restoration of dental health in orthodontic treatment were expected to be accompanied by the improvement of facial aesthetics [30]. Dental surgeons have been able to make virtual predictions and treatment planning with the help of 3-dimensional digital technology [22, 23, 31, 32]. Studies have shown that images of different dimensions affect the aesthetic perception of smiles [26–28]. Therefore, in order to make better use of 3-dimensional data to achieve the ideal therapeutic goals, it is particularly important to gain insight to the differences of aesthetic perception between traditional 2D photographs and the developing 3D dentofacial images.

When comparing the perceptual difference between population groups, no significant difference was found between orthodontists and postgraduate students in the aesthetic rating of smiles either with 2D or 3D images. However, it is worth noting that when evaluating 3D images, orthodontists rated the attractive smiles lower and rated the unattractive smiles higher compared with postgraduate students, indicating that orthodontists were more conservative in their use of 3D images. In addition, the questionnaires showed that postgraduate students had higher acceptance of 3D images and believed that 3D images could provide more details of smile features for diagnostic analysis. Dental students and senior orthodontists are important participants in the dental treatment. Understanding the characteristics of their perception of diagnostic data can help us optimize clinical decision. It can be inferred from the results of this study that postgraduates have a higher acceptance of 3D images and have earlier adaptation to 3D technologies [26, 28], resulting in a wider range of ratings. However, senior orthodontists have richer clinical experience, and their judgment on smile aesthetics is more comprehensive, leading to a narrower rating range. It is believed that with more contact and practices, senior orthodontists will be more proficient in 3D dentofacial images. Therefore, more research is needed to analyze the characteristics of their perception of 3D diagnostic data before 3D technologies are more widely used in clinical practice.

When it comes to the perceptual difference between image types, evaluators rated the 3D images lower than that of the 2D photographs. However, previous studies have shown the opposite, with 3D images received higher scores compared with 2D images [26, 27]. This may be attributed to the different types of 2D images selected. In the previous studies, in order to simulate and predict the outcome of restorative therapy, 2D photographs were integrated with 3D dental diagnostic waxing. Thus, the aesthetic perception to these kinds of integrated 2D images will be affected to some extent. In the present study, unintegrated 2D photographs were selected, aiming to compare the perceptual differences between traditional 2D photographs and 3D dentofacial images, which was intent for the aesthetic assessment scenarios of the moment before or after dental treatment.

The ICCs of retest consistency showed that the reproducibility of 3D dentofacial images for smile evaluation is lower than the 2D photographs, which may be one of the disadvantages of 3D evaluation. One of the possible reasons is that orthodontists and dental students have more experience with 2D facial images, which are used as traditional dental diagnostic data for decades. In contrast, they are relatively unfamiliar with the newly 3D dentofacial images and lack relevant experience of use. In addition, because 3D dentofacial images can provide more information about smile features, evaluators may need to process more information of smile esthetics. However, dental practitioners may not have established corresponding criteria for smile esthetics based on the additional information in 3D images, which may lead to the lower repeatability of esthetic evaluation. With the increasing use of 3D images in clinical practice and scientific research, the reproducibility of the 3D dentofacial images for smile evaluation may improve.

In order to further investigate the reasons of the aesthetic differences between records, the multiple linear regression showed that the commissures and facial profile were the main factors that resulting in the perceptual difference between 2D photographs and 3D dentofacial images. Previous studies have shown that commissures move in three dimensions [33]. There is a significant movement of the lips in sagittal dimensions during smiling, which is not observable in the front view [34]. In addition, previous studies have proven that the lateral and oblique views are also of great concern in smile evaluation [13, 14]. An increase protrusion of the mandible tends to draw attention from other facial features to the lower face when evaluating lateral views [35]. The inclination of teeth, protrusion of lips, and nasolabial angle are important factors that affect the smile attractiveness too [36]. Therefore, we can speculate that 3D dentofacial images can provide more diagnostic details in the aspect of the commissure movements and the profile of smile compared with traditional 2D photographs, which has potential applications in the diagnosis and treatment of facial paralysis [37], the treatment planning and outcome evaluation of facial plastic surgery, and the aesthetic evaluation in orthodontics. 3D dentofacial images can be used as supplementary data for the diagnosis and treatment planning of oral diseases and provide more diagnostic information for clinical decision-making.

In this study, patients after orthodontic treatment were selected to exclude the confounding factors on the aesthetic evaluation of smile such as crowding and tooth-loss. Unlike previous studies which changed smile characteristics quantitatively [26, 27], this study used numbers of real samples from clinical patients, which is more comparable to the real clinical application scenarios. Previous studies have shown that the three-quarter view receive high attention in aesthetic evaluation [13]. Many smile features have been neglected in frontal views solely, and the smile features in lateral and oblique views can be affected by different type of malocclusion [15]. Therefore, 2D photographs of frontal, oblique and lateral views were used in this study for the more comprehensive perceptual comparison between different dimensional records. Previous studies have indicated that evaluators have different preferences for smile morphological features under different viewing angles, such as the arc ratio, most posterior maxillary teeth visible, and mandibular teeth exposure [14]. The present study suggested that the commissure, smile arc, buccal corridor and facial profile received higher attention in 3D dentofacial images than in 2D photographs, while only the commissure and facial profile affect the aesthetic perception of the smile between dimensional records. This may be attributed to the fact the influence of different dimensions of smile morphology on smile aesthetics is inconsistent. Therefore, it becomes urgent to find out the correlation between the 3D morphology of smile and the smile aesthetic, and to establish the aesthetic standard of 3D smile reproductions, in order to make the 3D technology better serve the realization of the aesthetic goal of oral therapy.

One of the limitations of this study is the limited type of evaluators included. The present study focuses on the perceptual difference of smile aesthetic between traditional 2D photographs and 3D dentofacial images, which requires the evaluators to have a good understanding of the smile evaluation process. Since this study involved many specialized concepts of smile characteristics, which may beyond laypeople’s knowledge. In addition, laypeople have relatively less experience in 3D image technology. Therefore, this study did not include laypeople as evaluators. However, due to the differences in aesthetic perception between patients and dentists in the process of diagnosis and treatment, patients' views are crucial to obtain satisfactory treatment outcomes. It was reported that laypeople were more neutral in their choice of different dimensional smile records [26, 28]. Therefore, more elaborate experimental design are recommended to examine the perceptual characteristics of 3D smile images from the views of laymen.

## Conclusions

Orthodontists perceived smile aesthetics consistently to postgraduate students, while senior orthodontists were more conservative when evaluating 3D dentofacial images. 3D dentofacial images were rated lower than traditional 2D photographs in smile evaluation. Most of the smile features affect the aesthetic smile perception, except for the visible width of upper dentition and buccal corridor. The lower aesthetic rating of 3D dentofacial images can be attributed to the perceptual difference of commissure and facial profile.

Orthodontists should consider these smile perceptual patterns in the use of 3D dentofacial images for smile evaluation.

## Supplementary Information

**Additional file 1.** A: integration of digital dental casts into 3D facial image (cheek retractor). B: integration of 3D facial image (smile) with 3D facial image (cheek retractor). Red regions indicate the registration reference areas

**Additional file 2.** Animation of 3D dentofacial smile image

## Data Availability

The datasets used in this study are available from the corresponding author on reasonable request.

## Abbreviations

2D: Two-dimensional
3D: Three-dimensional
VAS: Visual analog scale
ICC: Intraclass correlation coefficient
