# Supplementary figures and images for: Perceptual difference of smile aesthetics between 2-dimensional photographs and 3-dimensional dentofacial images: a cross-sectional study
Source: BMC Oral Health. 2023 Feb 16;23:104. doi: 10.1186/s12903-023-02798-2 (PMC9933254; doi:10.1186/s12903-023-02798-2)

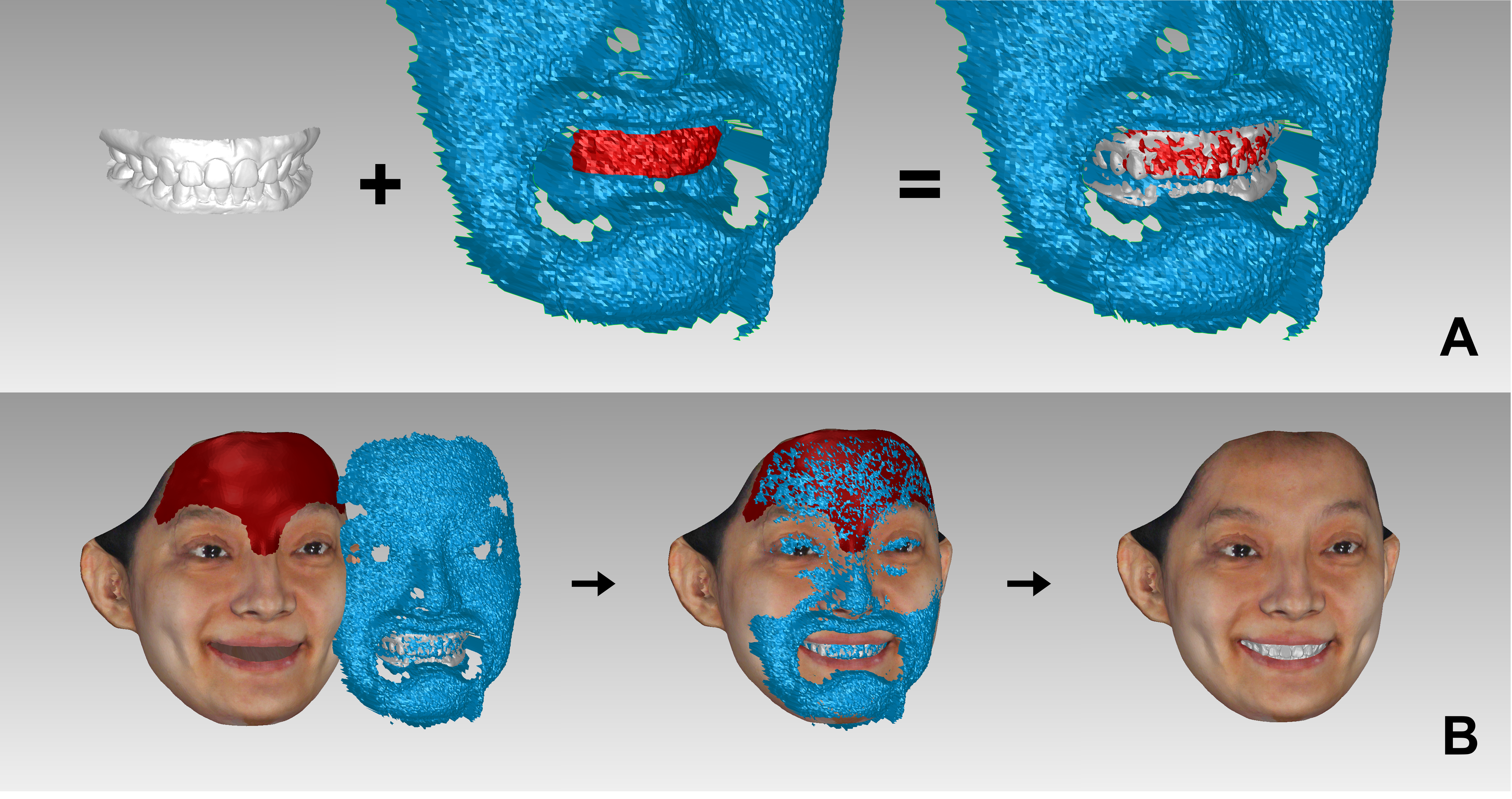

Supplement: Supplementary file 1 — Additional file 1. A: integration of digital dental casts into 3D facial image (cheek retractor). B: integration of 3D facial image (smile) with 3D facial image (cheek retractor). Red regions indicate the registration reference areas [file 12903_2023_2798_MOESM1_ESM.tif]
